# Self-Awareness in Survivors of an Acquired Brain Injury and Its Impact on Caregiver Burden

**DOI:** 10.3390/brainsci16040383

**Published:** 2026-03-31

**Authors:** Caleb Barcenas, Pamela Klonoff, Alexandra Theodorou, Jon Van Doren, Samuel Schaffer, Edward Koberstein, Joseph Murthy, Matty del Pino Luna, Santiago Palmer Cancel

**Affiliations:** 1 The Center for Transitional Neuro-Rehabilitation, Barrow Neurological Institute, St. Joseph’s Hospital and Medical Center, Phoenix, AZ 85013, USA; pamela.klonoff@commonspirit.org (P.K.); alex.theodorou@commonspirit.org (A.T.); samuel.schaffer@commonspirit.org (S.S.); edward.koberstein@commonspirit.org (E.K.); joseph.murthy@commonspirit.org (J.M.); matty.delpinoluna@commonspirit.org (M.d.P.L.); santiago.palmer-cancel@commonspirit.org (S.P.C.); 2 Department of Psychology, Adler University, Chicago, IL 60602, USA; jbvandoren@gmail.com

**Keywords:** self-awareness, caregiver burden, neurorehabilitation, brain injury, holistic milieu neurorehabilitation, acquired brain injury

## Abstract

**Highlights:**

**What are the main findings?**

- Self-awareness in survivors of an acquired brain injury (ABI) significantly predicted their caregivers’ level of burden.
- The time since injury for survivors of an ABI did not significantly predict their caregivers’ burden levels.

**What are the implications of the main findings?**

- Increasing self-awareness in survivors of an ABI through participation in outpatient holistic milieu neurorehabilitation predicts a decrease in caregiver burden.
- The time since injury for survivors of an ABI, up to the chronic phases of recovery, does not significantly predict their caregivers’ burden, which supports awareness training regardless of time since the ABI.

**Abstract:**

Background/Objectives: After an acquired brain injury (ABI), caregiver burden in family members is a clinical concern. Prior research has demonstrated that improved self-awareness in survivors of an ABI reduces caregiver burden. We examined the relationship between caregiver burden and ABI survivors’ levels of self-awareness across a span of injury chronicity following discharge from outpatient holistic milieu neurorehabilitation. Method: This retrospective observational study analyzed data on 59 individuals with heterogeneous ABIs who participated in an outpatient holistic milieu neurorehabilitation program from 2021 to 2025. This study utilized the discrepancy model of the Mayo-Portland Adaptability Inventory-4 (MPAI-4) to measure self-awareness in survivors of an ABI by calculating a discrepancy score from the self- and caregiver-rated MPAI-4 total score. Demographic information (age, education, race/ethnicity), injury history (injury type, age at injury, chronicity), program variables (length of program participation), functionality (MPAI-4), and caregiver burden (Zarit Burden Interview) at discharge were collected. Results: In order to predict caregiver burden based on self-awareness of an ABI survivor and time since injury, a multiple linear regression analysis was used. Although the multiple regression model significantly predicted caregiver burden, only self-awareness added significantly to the prediction and accounted for a modest proportion of the variance in caregiver burden. Conclusions: Self-awareness, as measured by utilizing the MPAI-4 discrepancy model, explained a modest proportion of the variance in caregiver burden regardless of time since injury. Among family members of survivors of an ABI, self-awareness of the survivor is a predictor of burden experienced by the family and would be beneficial to address as part of neurorehabilitation.

## 1. Introduction

Patients with an acquired brain injury (ABI) can experience a wide range of physical, cognitive, behavioral, and emotional difficulties [1,2,3,4]. A working definition of self-awareness is the understanding of one’s strengths and challenges and their functional implications following a brain injury [5]. Self-awareness is multidimensional and is affected by neurological, psychological, social, and cultural factors, as well as pre-injury attributes, neurocognition, and personal appraisals [5]. After an ABI, patients can often exhibit reduced ability to accurately perceive their deficits and understand how their deficits affect their daily functioning [6,7]. Lowered self-awareness has been linked to unrealistic expectations and goals [7,8,9], worse functional outcomes [7,10,11,12], worse neuropsychological performance [13,14,15], worse outcomes and poor compliance in rehabilitation [16,17], and reduced capacity to utilize compensatory strategies [9,18,19].

Self-awareness of deficits has been viewed on a continuum ranging from full awareness to a complete absence of awareness [20]. Reduced self-awareness can be explained by anatomic and functional changes in the frontal and parietal lobes in both neurodegenerative diseases and ABIs [21,22,23]. The executive dysfunction that survivors of an ABI experience can partially explain the increased frequency of maladaptive behaviors they engage in, which has also been shown to lead to significant distress in survivors’ loved ones [24,25].

The prevalence of lack of awareness of deficits, or impaired self-awareness after an ABI, ranges from 30% to 97% [12,26,27,28]. The frequency of impaired self-awareness is not well established due to variations in measurement methods and populations assessed [26,27,29]. There are several different approaches to determining the level of self-awareness, with the most prevalent involving interviews or using checklists to compare differences between how patients rate their post-injury challenges and how others, such as family members or medical providers, rate them [30]. Within these approaches, the most widely used is the computation of a discrepancy score by comparing self-ratings of competencies by patients and ratings by informants who are aware of the patients’ everyday functioning [31,32,33], although there is no gold standard [34]. Prior research has found that utilizing the Mayo-Portland Adaptability Inventory-4 (MPAI-4) Total Score to create a discrepancy score by subtracting patients’ self-ratings from close others’ ratings yields a high predictive value for caregiver burden [35].

Notably, determining the rate of reduced self-awareness is further complicated by other factors that researchers have identified over the course of rehabilitation. Based on the literature, one factor that affects self-awareness is the amount of time since injury [36,37,38,39]. The general consensus amongst researchers is that increased time since injury is associated with improved self-awareness [19,30,36,39,40,41,42], although these studies have assessed self-awareness only up to five years after the date of injury. Kelley [11] found that over the span of 17 years in patients with a traumatic brain injury (TBI), self-awareness continued to increase over 24 months from the initial date of injury, but then it appeared to plateau by five years, with indications that it did not change significantly beyond that period. In considering other potential contributing factors, Richardson [43] found that time since injury predicted self-awareness, whereas injury severity, cognition, and mood did not. As previously mentioned, reduced self-awareness of deficits in survivors of ABIs is associated with increased burden experienced by their close others, or individuals with whom they are close interpersonally.

Individuals with ABIs, including TBIs, brain tumors, and stroke, often experience a significant decline in their independence, rate of employment, productivity, and quality of life [11,44]. This overall decline in functioning is also associated with a greater dependency for activities of daily living [45,46,47]. In addition to formal care provided by medical professionals, after acute hospitalization for an ABI, many patients return home, where family members or close others must frequently provide care for them [48,49]. While informal care reduces the need for professional caregivers, it puts considerable demands on those closest to patients with an ABI [50].

Informal caregivers take on substantial social, physical, emotional, and financial burden [51,52]. The literature estimates that a significant burden has been reported in 25–54% of close others providing care to patients recovering from a stroke [53] and in 40–61% for those providing care to patients with a TBI [54,55,56]. The effect of caregiving on informal caregivers depends on the time spent, as well as the nature and severity of patients’ impairments [57]. Thus, the more assistance provided, the more burden experienced by close others [58]. Amongst the various issues that caregivers report encountering are patient mood problems, which are rated as the most stressful, along with difficulties with memory and bowel control [59].

Previous research has demonstrated that the close others of patients with an ABI are at a higher risk of depression [60], anxiety [61,62], reduced life satisfaction, and social isolation [63]. The relationship between patients with an ABI and their close others is bidirectional, as increased emotional distress in close others negatively affects patients’ responses to rehabilitation [59,64,65,66,67]. Lehan [68] found that survivors receiving care from a family member who reported a higher level of burden had poorer objective neuropsychological functioning. Similarly, lower levels of trait anxiety, burden, and strain in caregivers were significantly associated with higher-quality caregiving, as well as a willingness to spend more time providing care to survivors of an ABI [69].

In addition to assessing the relationship between close others’ burden and the amount of time spent providing caregiving assistance, researchers have also begun to investigate the effects of time since injury on caregivers’ experienced burden. Considering previous research, which suggests that increased time since injury is associated with improved self-awareness in survivors of an ABI, it could be assumed that close other burden would similarly improve with increased time since injury. However, the limited research on this topic remains inconsistent, and there has been a wide range of other variables identified that affect close other burden over the course of patients’ recovery.

Research has identified several factors that predict higher levels of burden one year after injury, including patients’ dysexecutive functioning, global disability, lack of social network, loneliness, and impaired functional status [50,70]. Beyond the first year after injury, some researchers have found that burden does not diminish within three to six years into the recovery phase [55], whereas others have found that the more time an individual has lived with an ABI (i.e., increased time since injury), the more burden their close others experience [71,72]. This is particularly relevant as the more burden informal caregivers experience, the less willing or able they are to provide care, thus increasing the symptoms that survivors of an ABI may exhibit [68].

Although there are many factors that contribute to close other burden, self-awareness has been found to be a significant predictor of burden [35,73]. Thus, neurorehabilitation programs have been designed to improve self-awareness and compensation use in patients with an ABI. More specifically, holistic, milieu neurorehabilitation programs address compensations for cognitive and functional deficits in patients with an ABI by offering emotional support, providing psychoeducation, and incorporating caregivers into the rehabilitation process, which may in turn help alleviate close other burden [5,74,75].

The current literature is limited in its exploration of how self-awareness impacts caregiver burden, particularly for individuals who are in the chronic phase of recovery following their ABIs and participate in outpatient holistic neurorehabilitation. The current study aimed to expand upon and explore the relationship between time since injury, close other burden, and the level of self-awareness of survivors of an ABI at the time of discharge from outpatient holistic milieu neurorehabilitation. We expected that increased time since the ABI would predict lower levels of close other burden. We also hypothesized that increased self-awareness would predict lower close other burden.

## 2. Materials and Methods

### 2.1. Participants

This retrospective observational study analyzed data on survivors of an ABI who participated in outpatient holistic milieu neurorehabilitation at the Center for Transitional Neuro-Rehabilitation (CTN), Barrow Neurological Institute, between 2021 and 2025. The sample included 59 survivor–close other pairs who completed one or more neurorehabilitation programs to facilitate: (a) home and community independence, (b) social relationships and quality of life, (c) work re-entry, and/or (d) school re-entry.

Demographic and injury-related characteristics of the sample appear in Table 1 and Table 2. ABI etiologies mainly included TBIs, strokes, and tumors, which reflects the typical heterogeneity in patients within this neurorehabilitation setting and other similar programs. Survivors of an ABI received therapies from Neuropsychology, Speech–Language Pathology, Occupational Therapy, Physical Therapy, Psychiatry, Recreational Therapy, Social Work, and Nutrition. Inclusion criteria were all patients with an ABI and their caregivers who participated in any of the CTN Programs, who were discharged between 2021 and 2025. Exclusion criteria were patients with discharge data sets that were incomplete (i.e., missing discharge data).

### 2.2. Procedure

The data analyzed in this study included questionnaires filled out by survivors of an ABI and their close others as part of standard care procedures at the time of admission and discharge.

### 2.3. Materials

The MPAI-4 was filled out by all 59 survivors of an ABI and by their close others. This 29-item questionnaire has been used as a primary outcome measure for patients with an ABI [76]. The questionnaire includes items that assess a range of physical, cognitive, emotional, behavioral, and social problems often experienced by survivors of an ABI, with a five-point scale ranging from 0 (None) to 4 (Severe Problem). It produces a total score as well as three subscale scores: Ability Index, Adjustment Index, and Participation Index. The total score and all three subscale scores are on a T-distribution, with higher scores indicating worse functioning. A measure of awareness was obtained by subtracting self-ratings of individuals with an ABI from close others’ ratings to create a discrepancy score. Positive discrepancy scores for the MPAI-4 questionnaire indicate less reporting of symptoms by individuals with an ABI compared with collateral ratings, assumed to be indicative of worse self-awareness. Discrepancy scores that are closer to zero or negative indicate greater reporting of impairments by the individual with an ABI than by the close other, which may suggest greater awareness and/or hypervigilance of deficits. Previous research has demonstrated good reliability and validity for all three of the subscale scores and the total index score [76,77].

The Zarit Burden Interview (ZBI) was completed by all close others of the survivors of an ABI. This self-report questionnaire is a 22-item, 5-point Likert scale (never = 0, nearly always = 4), with higher scores indicating greater burden. Total scores range from zero to 88 [78]. The ZBI is a reliable measure of caregiver burden, with a Cronbach α of 0.92 [79], and is frequently used with informal caregivers of patients with an ABI [80,81,82].

### 2.4. Data Analyses

All analyses were conducted using the IBM Statistical Package for the Social Sciences (SPSS) (v29). Participants were initially compared by demographic, injury-related, time since injury, and caregiver-related characteristics.

Pearson’s product-moment correlations were performed to examine the relationship between age, MPAI-4 Total Awareness Score, and caregiver burden; a normal distribution was not found for the variables in question. As such, Spearman’s rank-order correlations were performed, as shown in Table 3.

To determine whether there were significant differences in caregiver burden based on survivors’ or caregivers’ demographic and injury-related factors, comparisons were conducted across selected variables, as presented in Table 4. Independent-samples *t*-tests and one-way analyses of variance (ANOVAs) were conducted to examine caregiver burden in relation to the ABI survivors’ gender, ethnicity, caregiver gender, and caregiver relationship. Residual analyses were performed to test for the assumptions of the ANOVAs and *t*-tests. Outliers for each cell of the analyses were assessed by inspection of boxplots; normality was assessed using the Shapiro–Wilks normality test for each cell of the analyses; and homogeneity of variances was assessed by Levene’s test. A Kruskal–Wallis H test was conducted to examine caregiver burden in relation to injury type, as the assumptions of normality and homogeneity of variances were violated for the latter variable.

A multiple linear regression analysis was run to predict family burden from self-awareness of survivors of an ABI based on MPAI-4 Total discrepancy scores and time since injury. Using the most common approach for measuring self-awareness of deficits [83], a discrepancy score was calculated using the self and significant other/caregiver MPAI-4 total score. Previous research from the CTN has demonstrated that the MPAI-4 total score had the highest predictive value for the level of caregiver burden [35]. The predictors included in the regression model were selected on the basis of both theoretical and empirical justification, consistent with a multiple regression approach in which all predictors of theoretical interest are identified a priori and entered into the equation simultaneously. In accordance with established model-building principles, the decision to exclude additional clinical variables was informed not only by statistical non-significance, but also by evaluation of their theoretical importance in the existing literature. The preliminary analyses previously described revealed no statistically significant associations between caregiver burden and patient age, treatment duration, caregiver relationship, patient gender, patient ethnicity, caregiver gender, or injury type. This pattern of non-significant findings is consistent with the broader caregiver burden literature, in which demographic and injury-related variables have similarly failed to independently predict burden across multiple studies [84,85,86], indicating that neither the empirical nor theoretical conditions necessary to justify their inclusion as predictors were met in the present study. The modest proportion of variance explained by the model is consistent with the multifactorial nature of caregiver burden, which has not been fully accounted for even in substantially larger studies, and reflects the inherent complexity of the outcome rather than omission of relevant predictors [85]. Data on injury severity were not available in the archival database and, therefore, could not be considered for inclusion. There was linearity, as assessed by partial regression plots and a plot of studentized residuals against the predicted values. There was independence of residuals, as assessed by a Durbin–Watson statistic of 1.659. There was homoscedasticity, as assessed by visual inspection of a plot of studentized residuals versus unstandardized predicted values. There was no evidence of multicollinearity, as assessed by tolerance values greater than 0.1. There were no studentized deleted residuals greater than ±3 standard deviations, no leverage values greater than 0.5, and no values for Cook’s distance above 1. The assumption of normality was met, as assessed by a Q-Q Plot.

## 3. Results

Table 1 and Table 2 summarize demographic information for the 59 survivors of an ABI and their close others. Overall, most were White/Caucasian (64.4%) males (71.2%) with an average age at injury of 35.2 years (SD = 16.06 years) and a mean of 14.75 years of education (SD = 2.4 years). The mean time since injury was 37.05 months (SD = 45.83), and the mean duration of treatment was 11.22 months (SD = 5.11). The primary etiologies of the ABIs for the patients were TBIs (40.7%) and brain tumors (32.2%), with a smaller percentage having sustained strokes (16.9%). Of the close others, the majority were spouses (52.5%). Of the close others, 81.4% self-identified as female.

### 3.1. Caregiver Burden in Relation to Characteristics of Survivors of an ABI

As shown in Table 3, Spearman’s rank-order correlations for caregiver burden across various demographic and injury-related variables revealed a statistically significant, positive correlation between age and education (ρ = 0.614, *p* < 0.01), as well as a positive correlation between treatment duration and time since injury (ρ = 0.539, *p* < 0.01). No statistically significant correlations emerged between the burden and self-awareness scores and the demographic and injury-related variables (*p* > 0.05).

### 3.2. Caregiver Burden in Relation to Demographic and Injury-Related Variables

Table 4 presents the values of caregiver burden between different groups. Overall, there were no statistically significant differences in mean caregiver burden scores between patient gender, *M* = 4.13, *t*(57) = 1.28; patient ethnicity, *M* = 12.76, *F*(3, 55) = 1.42; close other relationship, *M* = 12.76, *F*(4, 54) = 0.42; or close other gender, *M* = 4.66, *t*(56) = −1.26. The results of the Kruskal–Wallis H test revealed that the distributions of caregiver burden scores were not similar for all injury types, as assessed by visual inspection of a boxplot. Thus, only mean rank scores are reported for injury type in Table 4. Mean rank caregiver burden scores were not statistically significantly different between injury types, χ^2^(3) = 1.21.

### 3.3. Predicting Family Burden from Self-Awareness of Survivors of an ABI and Time Since Injury

As shown in Table 5, the multiple regression model statistically significantly predicted close other burden, *F*(2, 56) = 5.346, *p* < 0.008, and adj. *R*^2^ = 0.130. While time since injury did not add statistically significantly to the model (B = 0.006, 95% CI [−0.002, 0.015], *p* > 0.05), self-awareness (MPAI-4 Total Awareness Score) added statistically significantly to the prediction (B = 0.064, 95% CI [0.023, 0.106], *p* < 0.004). The adjusted *R*^2^ for the overall model was 13.0%, a small effect size according to Cohen (2013) [87], indicating that the model explained a relatively modest proportion of the variance in caregiver burden. The regression coefficients can be found in Table 5 (below).

## 4. Discussion

The current study examined the relationship between time since injury, caregiver burden, and self-awareness of patients with an ABI at the time of discharge from outpatient holistic milieu neurorehabilitation. Discrepancies between patients’ and close others’ reported level of functioning are commonly used as an index of self-awareness in patients with an ABI [14,40,69,88,89], and in this study, the MPAI-4 total score was used to assess self-awareness. While many researchers have utilized a categorical approach with predetermined thresholds (i.e., impaired vs. unimpaired) to define self-awareness as assessed with MPAI-4 discrepancy scores, it has been argued that, beyond the use of arbitrary thresholds, the use of categorization may be an oversimplification of a complex phenomenon [30]. Therefore, this study used a non-categorical approach, where patients’ self-awareness was evaluated as a continuous measurement.

In the present study, it was expected that patients’ level of self-awareness would predict caregiver burden. Consistent with previous research, the level of patients’ self-awareness was shown to be a significant predictor of close other burden [35,73]. Previous research has demonstrated that individuals with an ABI tend to overestimate their functional capacities, engage in unsafe behaviors, and require more supervision and assistance from close others for activities of daily living, thus leading to increased distress experienced by their close others [11,73]. It stands to reason that close others would experience increased stress when ABI survivors with reduced self-awareness engage in unpredictable behaviors, including dangerous or high-risk behaviors. Further, Chesnel [90] found that subjective burden perceived by close others was associated with patients’ level of self-awareness, whereas patients’ functional independence, global disability, and return to work were not. While the results are statistically significant, the observed effect size suggests that self-awareness explains a relatively small proportion of the variance in caregiver burden. This highlights the complex nature of caregiver burden and the need for future studies to explore other factors that influence the overall level of caregiver burden.

Contrary to our hypothesis and previous studies [11,30,39,40], there was no evidence that increased time since injury was associated with improved self-awareness. One explanation for this is that many previous studies assessed self-awareness much earlier (e.g., five years or less) in the recovery course [30,37,40,91], while the current study included patients who were more than 12 years post-injury. Previous research examining the relationship between injury severity and time after injury is mixed, as some studies have found both to be significant predictors of self-awareness [36,37,39,92,93,94,95], whereas others have not [14,27,31,45,96,97]. A plausible explanation for the absence of a relationship between time since injury and patients’ self-awareness in the current study is the nature of holistic milieu neurorehabilitation, which incorporates intensive outpatient therapies specifically aimed at enhancing self-awareness. Consequently, patients who underwent neurorehabilitation at the CTN may have experienced an increase in self-awareness, thereby mitigating the influence of time since injury. Although injury severity was not a variable in this current study, the overwhelming majority of studies examining injury severity and time since injury included only patients who had sustained traumatic brain injuries, thus excluding patients with other ABIs such as stroke, brain tumor, encephalitis, anoxic injuries, or viral infections. It is possible that including a heterogeneous sample of various types of ABIs may account for at least some of the differences in the findings from the current study compared to previous research.

Contrary to our hypothesis and results from previous studies [48,53,61,98], the current study did not find evidence that time since injury was associated with decreased close other burden. There are several studies that have yielded conflicting results, including the finding that there was no change in burden over time [99], as well as the finding indicating slight increases in burden over time [100], although both studies examined changes only one year after injury. One distinctive aspect of this study is the heterogeneity in the ABI sample. While the mean caregiver burden scores did not significantly differ between the various caregiver and patient demographic and injury characteristics, a larger sample size would effectively increase the power to detect differences in a heterogeneous ABI sample. Additionally, further exploration of other factors, such as premorbid comorbidities, pre-injury psychological status, and coping styles, is warranted, as prior studies have identified them as contributing factors to caregiver burden [86,101].

One possible explanation for the lack of significant findings between time since injury and close other burden in the current study could be related to the nature of the role of the close other in the patient rehabilitation process. Specifically, previous studies examining burden have typically used patients’ closest relatives who serve as the patient’s primary informal caregiver, which is defined as the person most responsible for day-to-day decision making and care of the patient [102]. Alternatively, it could be possible that the lack of findings between time since injury and close other burden is due to factors not considered in this study.

Previous research has shown that the level of burden reported by family members of individuals with a TBI was significantly higher for family members who reported spending more time taking care of and supervising the patient [55]. Interestingly, the burden reported by close others is more strongly associated with the responsibilities of caregiving than with whether or not the caregivers lived with the patients [58]. Future studies should account for objective measurement of the amount of time close others spent providing care, as well as the type of caregiving responsibilities they provide. Even though this present study did not account for these variables, the close others who participated were considered to have a close interpersonal relationship with the patient and provided a range of assistance, sometimes related to self-care or instrumental activities of daily living and, other times, related to distant supervision (e.g., double-checking patients’ finances). Further, the approach for assessing the burden reported by family members or individuals who were interpersonally close to patients with an ABI is consistent with previous studies [35,55,73,103].

### Limitations

The use of the discrepancy model between survivors of an ABI and their close others to evaluate self-awareness has some limitations due to the need for subjective reporting from the perspective of close others. It is possible that caregivers who experience higher levels of burden are likely to consider their loved ones as more impaired functionally, regardless of their true functional levels [73], resulting in a biased discrepancy score, thus suggesting an artificially reduced level of self-awareness. Given the cross-sectional nature of our data, causal inferences regarding the impact of neurorehabilitation should be interpreted with caution. The regression model explained a relatively modest proportion of the variance in caregiver burden, indicating that while self-awareness and time since injury together produced a statistically significant model, they account for only a small portion of the factors contributing to close other burden. This underscores the multifactorial nature of caregiver burden and suggests that variables not captured in the present study, such as premorbid psychological status, coping styles, social support, and the nature and intensity of caregiving responsibilities, may contribute meaningfully to close other burden and warrant exploration in future research. This study was conducted using standard-of-care discharge questionnaires following participation in an outpatient holistic milieu neurorehabilitation program that emphasizes caregiver support and self-awareness in survivors. Due to the specific nature of this program, it is uncertain if these results can be generalized to traditional outpatient neurorehabilitation.

## 5. Conclusions

In summary, in an outpatient holistic milieu program, at the time of discharge, increased levels of self-awareness in patients with an ABI significantly predicted a modest proportion of the variance in caregiver burden, as reported by close others, whereas time since injury did not. Future studies should examine the degree to which changes in patients’ self-awareness over the course of their neurorehabilitation predict changes in close other burden over the same period of time, as well as assess the potential effects on various outcome variables, such as rates of employment, openness to compensation use, and reduction in problematic or risk-taking behavior. Additionally, researchers assessing the level of self-awareness in survivors of an ABI have also compared staff ratings of patient functioning with self-ratings of functioning to measure patients’ level of self-awareness on the MPAI-4 [104]. It would be beneficial for future studies to begin assessing which discrepancy scores are more predictive of close other burden and patients’ level of functioning. Given that time since injury does not significantly predict caregiver burden, therapeutic interventions to increase self-awareness in neurorehabilitation settings can be implemented regardless of the amount of time that has elapsed from injury occurrence to when a survivor of an ABI engages in neurorehabilitation. Furthermore, a program that emphasizes increasing survivors’ self-awareness about their strengths and challenges and how these manifest in the community can provide a strong foundation for decreasing caregiver burden.

## Figures and Tables

**Table 1:** Demographic information for the sample.

Characteristic	M	SD	Range
Education (in years)	14.75	2.40	12–20
Age at discharge (in years)	38.39	14.51	19–68
Age at injury (in years)	35.17	16.06	7–66
Time since injury (in months)	37.05	45.83	4–209
Treatment duration (in months)	11.22	5.11	2–20

Note. n = 59. M = mean; SD = standard deviation. Time since injury = the amount of time between the date of a patient’s injury and the date of their discharge from the CTN, as measured in months.

**Table 2:** Demographic information for the sample.

	*n*	Percentage
Gender		
Male	42	71.2
Female	17	28.8
Ethnicity		
White/Caucasian	38	64.4
Hispanic/Latino	12	20.3
Other/Biracial	5	8.5
Black	4	6.8
Type of Injury		
TBI	24	40.7
Tumor	19	32.2
Stroke	10	16.9
Other Neurological (i.e., encephalitis, anoxic injuries, or viral infections)	6	10.2
Close Other Relationship		
Spouse	31	52.5
Parent	22	37.3
Sibling	2	3.4
Significant Other	2	3.4
Other (i.e., child or friend)	2	3.4
Close Other Gender		
Female	48	81.4
Male	11	18.6

**Table 3:** Spearman’s rank-order correlations between caregiver burden and demographic- and injury-related variables.

Variable	1	2	3	4	5	6
1. Age	1.00					
2. Education	0.614 *	1.00				
3. Treatment duration (months)	−0.029	−0.134	1.00			
4. Time since injury (months)	−0.111	−0.248	0.539 *	1.00		
5. ZBI Total	−0.006	0.189	0.150	0.202	1.00	
6. MPAI-4 Total Awareness Score	0.047	0.133	0.040	−0.099	0.217	1.00

Note. n = 59. MPAI-4 = Mayo-Portland Adaptability Inventory-4; ZBI = Zarit Burden Interview. * *p* < 0.01.

**Table 4:** Comparison of demographic and injury-related variables based on the ZBI total score.

Variable	M (SD)	*p*-Value
Patient Gender		0.21
Male	13.95 (12.39)	
Female	9.82 (7.59)	
Patient Ethnicity		0.25
White/Caucasian	14.92 (12.52)	
Hispanic/Latino	8.42 (6.24)	
Other/Biracial	7.80 (12.44)	
Black	11.50 (3.87)	
Type of Injury		0.75
TBI	27.25	
Tumor	30.55	
Stroke	33.00	
Other Neurological (i.e., encephalitis, anoxic injuries, or viral infections)	30.58	
Caregiver Relationship		0.80
Spouse	13.58 (13.11)	
Parent	12.64 (9.76)	
Sibling	4.50 (3.54)	
Significant Other	15.00 (4.24)	
Other (i.e., child or friend)	7.50 (3.54)	
Caregiver Gender		0.21
Male	8.64 (7.42)	
Female	13.30 (11.67)	

Note. n = 59. ZBI = Zarit Burden Interview; TBI = traumatic brain injury; M = mean; SD = standard deviation.

**Table 5:** Multiple linear regression for the ZBI.

	B	95% CI for B		SE B	β	R^2^	ΔR^2^
		LL	UL				
Model						0.160	0.130 *
MPAI-4 Total Awareness Score	0.064 *	0.023	0.106	0.19	0.381 *		
Time since injury	0.006	−0.002	0.015	0.004	0.180		

Note. n = 59. MPAI-4 = Mayo-Portland Adaptability Inventory-4; ZBI = Zarit Burden Interview; CI = confidence interval; LL = lower limit; UL = upper limit; SE B = standard error of the coefficient; β = standardized coefficient; R^2^ = coefficient of determination; ΔR^2^ = adjusted R^2^. * *p* < 0.05.

## Data Availability

The data presented in this study are available on request from the corresponding author due to privacy reasons.

## Abbreviations

The following abbreviations are used in this manuscript:

ABI	Acquired brain injury
ANOVA	One-way analysis of variance
CTN	Center for Transitional Neuro-Rehabilitation
MPAI-4	Mayo-Portland Adaptability Inventory-4
SPSS	Statistical Package for the Social Sciences
TBI	Traumatic brain injury
ZBI	Zarit Burden Interview
